# Age-Related Success with Elective Single versus Double Blastocyst Transfer

**DOI:** 10.5402/2011/656204

**Published:** 2011-11-17

**Authors:** Brooke E. Friedman, Lynn B. Davis, Ruth B. Lathi, Lynn M. Westphal, Valerie L. Baker, Amin A. Milki

**Affiliations:** ^1^Stanford Fertility and Reproductive Medicine Center, Stanford University Medical Center, 900 Welch Road, Suite 350, Palo Alto, CA 94304, USA; ^2^Seattle Reproductive Medicine, 1505 Westlake Avenue, North Suite 400, Seattle, WA 98109, USA

## Abstract

*Background.* Although the optimal outcome of assisted reproductive technology (ART) is a healthy singleton pregnancy, the rate of twin gestation from ART in women over the age of 35 is persistently high. *Methods/Findings.* We compared clinical pregnancy rates (PRs), ongoing pregnancy/live birth rates, and multiple gestation rates (MGRs) in 108 women who chose elective single blastocyst transfer (eSBT) to 415 women who chose elective double blastocyst transfer (eDBT) at a hospital-based IVF center. There was no significant difference in PR between eSBT and eDBT (57.4% versus 50.2%, *P* = 0.47) nor between eSBT and eDBT within each age group: <35, 35–37, 38–40, and >40. The risk of multiple gestations, however, was greatly increased between eSBT and eDBT (1.6 versus 32.4%, *P* < 0.00005), and this difference did not vary across age groups. *Conclusion(s).* Women undergoing eDBT are at uniformly high risk of multiple gestation regardless of age. eSBT appears to significantly lower the risk of multiple gestation without compromising PR.

## 1. Introduction

The optimal outcome of assisted reproductive technology (ART) is a healthy singleton pregnancy [1]. The rate of twin gestation from ART in women over the age of 35, however, remains stable at 30%, and multiple gestations comprise a quarter of all live births from fresh, nondonor cycles among women aged 38–40 [2]. 

In 1998, the Society for Assisted Reproductive Technology (SART) and the American Society of Reproductive Medicine (ASRM) issued the first in a series of guidelines regarding the optimal number of embryos to be transferred based on patient age [3]. Aimed at reducing rates of high-order multiple (HOM) pregnancies, these guidelines were revised most recently in 2006 and recommend transferring two to three cleavage stage embryos for women aged 35–37, and no more than two blastocysts, depending on prognostic factors and patient preference. For women aged 38–40 years, the recommendation is for three to four cleavage stage embryos, and between two to three blastocysts [4]. The implementation of these guidelines has been associated with a decrease in HOM [5], but the rate of twins remains persistently high. 

An extensive body of literature has demonstrated that twin gestations carry increased risk of adverse outcomes, such as prematurity, low birth weight, infant mortality [6, 7], and maternal mortality [8]. Twin gestations also compound the health threats to women of advanced maternal age, who are already at increased risk of developing complications such as gestational diabetes [9] and preeclampsia [10]. For example, women over the age of 35 with preeclampsia have three times the risk of pregnancy-related mortality than their younger counterparts [11]. These adverse outcomes have had a considerable impact on public health and many have called for policy change to decrease the prevalence of multiple gestation.

Given that the transfer of multiple embryos increases the risk of a multiple gestation, many have suggested a move towards elective single embryo transfer (eSET), although this recommendation has been focused mainly on women less than age 36 [12]. Several randomized controlled trials in Europe have demonstrated that eSET significantly diminishes twin gestations and yields comparable live birth rates in good prognosis patients [12, 13].

A handful of European studies have also addressed the role of eSET in the advanced maternal age population. One study found that eSET could be safely applied to patients aged 36–39, while dramatically decreasing the multiple gestation rate (MGR) and achieving similar pregnancy rates (PRs) [14]. Another study found that eSET could be offered to women younger than 38 in the first three IVF treatment cycles without compromising PR, although the mean patient age in this study was 32.5 years [15]. 

While these studies analyzing eSET have focused primarily on day 3 embryo transfer, blastocyst transfer (BT) has also emerged as a potential approach to reducing MGR as generally fewer embryos are transferred. Several studies have shown superior outcomes with BT compared to cleavage stage embryo transfer [16–21]. The superior implantation rate seen with BT has mainly been attributed to better embryo selection. 

A recent randomized prospective controlled trial found higher PR with elective BT over the elective transfer of a cleavage stage embryo [22]. This study, however, focused only on women 36 years of age or less. Due to the paucity of data regarding eSBT in the advanced maternal age population, we previously reviewed our preliminary experience with eSBT in women older than 35 and found that PR was quite promising, with half of the patients experiencing an ongoing pregnancy or live birth [23]. More limited literature exists comparing the elective single blastocyst transfer (eSBT) with elective double blastocyst transfer (eDBT) between the ages of 35 to 40. Although the inclusion criteria in these studies allowed patients up to age 37 [24], 38 [25] or 39, [26], the focus tended to be on younger women. 

As described previously, women of advanced maternal age are at higher risk of maternal and perinatal adverse outcomes with twin gestation. It is with these patients that physicians tend to be more aggressive with number of embryos transferred as age has been demonstrated to be a powerful negative predictor of IVF success [2]. Our hypothesis was that among patients with blastocysts available for cryopreservation, irrespective of advanced maternal age, eSBT would yield comparable pregnancy rates to eDBT, while minimizing the risks of a multiple gestation. Therefore, our aim was to compare pregnancy outcomes between eSBT and eDBT, stratified by age. We were able to achieve this aim by utilizing the methods described below.

## 2. Materials/Methods

All fresh nondonor cycles in which patients electively chose to transfer one or two blastocysts at the Stanford University IVF program over a five-year period were analyzed. The following ovarian stimulation protocols were used: luteal down regulation (long), antagonist protocol, and microdose leuprolide acetate (flare), as previously described [27]. Human chorionic gonadotropin (hCG) was generally administered when at least two follicles reached an average diameter of >17 mm. Transvaginal ultrasound-guided oocyte retrieval was performed 35 hours after hCG administration. BT was offered to patients with 3 or more 8-cell embryos on day 3. 

Prior to the start of their cycle, patients attended a group IVF informational seminar, in which the advantages and disadvantages of single versus multiple embryo transfer for different age groups were covered in detail. A preliminary decision on the number of embryos to transfer was made at the time of the patient's initial IVF physician consultation, where existing data from the literature and from our own program regarding success rates and the incidence of multiple gestation were further discussed, with emphasis on the fetal and maternal risks of twin pregnancies. The final decision regarding how many blastocysts to transfer was ultimately made by the patient at the time of transfer. 

Sequential culture with P1/Blastocyst Medium (Irvine Scientific, Santa Ana, Calif) or Quinn's Advantage Cleavage/Blastocyst Media (SAGE In-Vitro-Fertilization, Inc., Trumbull, Conn) was used. The decision of which blastocyst to transfer was based on the definition of the inner cell mass and trophectoderm and the degree of expansion, with the remaining blastocysts cryopreserved on day 5 or 6 as previously described [28].

Ultrasound-guided embryo transfer was performed using a Tefcat or Echotip Softpass catheter (Cook Ob/Gyn, Spencer, Ind). Serum quantitative ß-hCG levels were obtained at 8–10 days after embryo transfer. A clinical pregnancy was defined as the presence of a fetal sac by transvaginal ultrasound. An ongoing pregnancy was defined as an intrauterine pregnancy continuing past 14 weeks gestation. 

Data collected in this study include age, number of oocytes retrieved, number of embryos, number of blastocysts cryopreserved, use of intracytoplasmic sperm injection (ICSI) and preimplantation genetic diagnosis (PGD), clinical PR, live birth/ongoing PR, MGR, cesarean section rate, and birthweight. Patient characteristics and cycle outcomes of the eSBT and eDBT groups were compared using *t*-test or Chi-square test for normally distributed variables and Wilcoxon rank-sum test for nonnormally distributed variables. Variables were tested for normality by the Kolmogorov-Smirnov test and those that were not normally distributed were logarithmically transformed. Statistical significance was determined as *P* < 0.05. Age was analyzed as both a continuous and a categorical variable (<35 years, 35–37 years, 38–40 years, and >40 years). We used one-way ANOVA to test differences between age groups and adjusted for multiple comparisons with the Scheffé multiple-comparison test. A generalized linear model was used to adjust for the potential confounder of age and number of embryos transferred. Statistical analyses were performed using Stata 9 (StataCorp LP, College Station, Tex). 

The study was approved by the Stanford University Research Compliance Office for Human Subjects Research (Institutional Review Board). Written consent was not obtained from patients because this was a retrospective chart review, with the study design conceived after the patients' treatments had been completed. Per the Stanford Institutional Review Board (IRB), an investigator can submit a proposal for studying the treatment outcomes of past patients whose treatments have been completed. It is not routine to obtain prior consent of all patients utilizing assisted reproductive technology for the possibility of using their outcome data in a future study. Given that this study was a retrospective chart review, the Stanford IRB granted a waiver of consent. Every reproductive endocrinology and infertility clinic in the United States must maintain outcome data on assisted reproductive technology treatments because this data must be reported to a nationally maintained database. All data for our study was carefully deidentified and personal health information removed prior to analysis. 

## 3. Results

Pregnancy outcomes in 108 women who chose eSBT were compared to 415 women who chose eDBT. The mean age of patients undergoing eDBT (35.0 years) was significantly higher than the mean age of patients undergoing eSBT (33.9 years) (*P* < 0.006) (see Table 1). There were no differences between the eSBT and eDBT groups in terms of prior live births or cycle characteristics, such as number of oocytes, number of embryos, number of blastocysts, and percent of cycles using ICSI or PGD. 

There was no significant difference in live birth/ongoing PR between the eSBT (48.1%) and eDBT (41.7%) groups (*P* = 0.22) overall. The live birth/ongoing PR was similar for eSBT and eDBT within each age group as well. Additionally, we found no significant differences in live birth/ongoing PR or MGR when comparing the different age groups (<35 years, 35–37 years, 38–40 years, and >40 years) (see Table 2). Logistic regression revealed no significant difference in live birth/ongoing PR, controlling for number of embryos transferred (OR 1.25, 95% CI 0.81–1.91) or for age (OR 0.96 per year, 95% CI 0.91–1.01). 

The MGR was 32.4% for women who underwent eDBT, compared with 1.6% in women who underwent eSBT (*P* < 0.00005). The one patient in the eSBT group with a multiple gestation was less than 35 years of age and had monozygotic twins. Among the eSBT and eDBT groups, there were two cases of viable monozygotic twinning out of a total of 269 clinical pregnancies (0.7%). All multiple gestation births were twins; there were no higher-order multiple births. 

There were, however, two patients in the eDBT group who initially had three viable fetuses on first trimester ultrasound. One patient initially had three separate gestational sacs of different sizes, each containing a viable fetus, and reported having intercourse four days prior to her eDBT. The other patient had two gestational sacs, with one viable singleton as well as viable monozygotic twins. Both these patients underwent multifetal pregnancy reduction and delivered twins and a singleton, respectively. 

There was no significant difference in mode of delivery between the eSBTs and eDBT groups. Mean birthweight was significantly smaller in the eDBT group (2832.2 g) compared with the eSBT group (3226.2 g) (*P* = 0.001). The mean birthweight of twin gestations (2369.7 g) was lower than the mean birthweight of singletons (3132.0 g) (*P* < 0.0001). There was no significant difference in the mean birthweights of singletons resulting from eDBT (3078.7 g) and from eSBT (3285.7 g) (*P* = 0.11). The 18.8% (19/101) of singletons that were known to begin as a vanishing twin gestation, however, had a significantly lower mean birthweight (2794.4 g) compared to singletons that began as such (3188.2 g) (*P* = 0.04). 

## 4. Discussion

In many European countries eSET is mandated by the government and has become a part of routine clinical practice [29]. In the United States, with most patients facing considerable out-of-pocket costs per IVF cycle, many patients and physicians are hesitant to move towards the transfer of a single embryo, for fear of compromising PR. Our results show, however, that when additional blastocysts are available for cryopreservation, eSBT can achieve comparable PR to eDBT across differing age groups, up to age 38. In our study, eSBT also appeared to be a promising option for women over the age of 38, although the analysis of PR in this age group is limited, as there were fewer eSBTs in this cohort, with only one patient in the >40 year old group who transferred a single blastocyst. Importantly, however, the MGR was approximately 30% in all age groups undergoing eDBT, suggesting that advanced maternal age does not protect against multiple gestations in this good prognosis group. 

Given the relatively small number of women over the age of 38 in our study, it is difficult to advocate a routine policy of single blastocyst transfer in this age group. Our data are encouraging, however, regarding the option of eSBT for older patients with blastocysts available for cryopreservation, who prefer to avoid a multiple gestation. These results are in accordance with the recent findings of Mullin et al., who also suggested that women under the age of 40 can choose eSBT as opposed to eDBT without reducing PR [26].

Despite the more limited numbers in our older cohort, this study has the largest eSBT cohort used to compare eSBT with eDBT in the United States and may provide reassurance to physicians who are reluctant to offer eSBT to women of advanced maternal age. Clearly, eSBT must be applied selectively to a good prognosis cohort. Randomized prospective trials have demonstrated that, in an unselected patient population, eSET can significantly compromise PR [30]. Our results suggest, however, that age alone should not automatically prevent inclusion in a good prognosis cohort. 

Among women with blastocysts available for cryopreservation, age does not appear to confer a significantly worse prognosis, as PR was comparable up to age 38. Although a larger proportion of younger women will have good quality blastocysts, those older women with blastocysts available for cryopreservation should not be excluded from the option of eSBT, particularly as they are at substantial risk of MGR with eDBT. 

Among MGRs resulting from ART, studies have demonstrated an increased risk of MZ twinning, with some suggesting added risk for MZ twinning with BT [31]. We have previously reported that with increased experience in blastocyst culture and transfer, our overall blastocyst MZ twinning rate decreased to within range of that seen with cleavage stage transfer [32]. In this current study, our rate of MZ twinning is double the rate reported for MZ twinning in nature (0.4%) [33] but is in accordance with the MZ twinning rates described in other retrospective series [34]. 

Interestingly, despite the higher proportion of twin gestations in the eDBT group, there were similar rates of cesarean delivery between the two groups. The high percentage of multiple gestations among the patients who underwent eDBT contributed to the lower birth weight seen in this group, as twin gestations are known to be at higher risk of preterm delivery and low birth weight. Another contributing factor to the birth weight discordance appears to be the presence of vanishing twins in the eDBT cohort. Among the singletons born in the eDBT group, the survivors of a vanishing twin gestation had a significantly lower mean birth weight, in keeping with previous studies [35, 36]. Thus, even if eDBT ultimately yields a singleton gestation, there may be adverse implications for the pregnancy through the implantation of more than one embryo. Physicians may wish to consider this as well in deciding between eSBT and eDBT. 

Due to the retrospective nature of the study, uncontrolled factors may have made the eDBT group less likely to conceive than the eSBT group, resulting in patient and physician decision to transfer an additional embryo. It is important to note, however, that there were no differences in percentage of patients with a prior live birth or mean number of oocytes, embryos, or blastocysts between the eSBT and eDBT groups, suggesting that overall these two cohorts comprised similar populations. 

The main strength of our study is that it presents the largest eSBT cohort in the United States, in order to compare eSBT with eDBT. Moreover, it is the most inclusive study on this topic, as most other authors have limited their study population to women less than 38 years of age, with significantly younger mean age. Larger studies are warranted, however, as our numbers of women over the age of 38 are modest. 

More studies, ideally prospective and randomized, are needed to further investigate the role of eSBT in older women with blastocysts available for cryopreservation, as women of advanced maternal age are at particularly high risk of complications associated with multiple gestations. Preliminary results suggest that good prognosis patients undergoing BT, including those of advanced maternal age, who wish to avoid a multiple gestation should transfer a single blastocyst, reassured that PRs do not seem to be significantly compromised. 

## 5. Conclusion

Women undergoing eDBT are at uniformly high risk of multiple gestation regardless of age. eSBT appears to significantly lower the risk of multiple gestation without compromising PR.

## Figures and Tables

**Table 1:** Patient characteristics and cycle outcomes.

	eSBT (*n* = 108)	eDBT (*n* = 415)	*P *value
Age, mean [SD]	33.9 [3.2]	35.0 [3.6]	0.0006^a^
Prior live birth (%)	28/108 (25.9)	76/339 (18.3)	0.08
Oocytes, mean [SD]	17.0 [6.3]	17.2 [6.5]	0.58
Embryos, mean [SD]	12.4 [5.8]	12.2 [4.9]	0.84
Blastocysts, mean^d^ [SD]	6.5 [3.8]	6.2 [3.0]	0.77
ICSI (%)	50/108 (46.3)	194/415 (46.7)	0.80
PGD (%)	9/108 (8.3)	25/415 (6.0)	0.28
Clinical PR (%)	62/108 (57.4)	207/415 (49.9)	0.16
Live birth/ongoing PR (%)	52/108 (48.1)	173/415 (41.7)	0.22
MGR (%)	1/62 (1.6)	67/207 (32.4)	0.00^b^
Cesarean rate (%)	12/38 (31.6)	61/161 (37.9)	0.47
Birthweight, mean (g)	3226.2	2832.2	0.001^c^

^a^ *P* < 0.01: *t*-test.

^b^ *P* < 0.00005: chi-square test.

^c^ *P* < 0.005: *t*-test.

^d^Blastocysts: mean includes blastocysts cryopreserved and transferred.

**Table 2:** Cycle characteristics and outcomes by age category.

****	<35 years		35–37 years		38–40 years		>40 years	
****	eSBT (*n* = 63)	eDBT (*n* = 208)	eSBT (*n* = 34)	eDBT (*n* = 113)	eSBT (*n* = 10)	eDBT (*n* = 81)	eSBT (*n* = 1)	eDBT (*n* = 13)
Oocytes, mean	16.7	17.4	17.5	17.9	16.3	15.7	23.0	17.5
Embryos, mean	12.6	12.3	12.5	12.5	11.1	11.4	13.0	13.3
Blastocysts, mean^e^	6.6	6.6	6.7	6.3	5.6	5.4	4.0	5.9
Cryopreserved blastocysts, mean	5.6	4.6	5.7	4.3	4.6	3.4	3.0	3.9
ICSI (%)	36/63 (57.1)	119/208 (57.2)	13/34 (38.2)	39/113 (34.5)	0/10 (0)^a^	29/81 (35.8)^a^	1/1 (100)	6/13 (46.1)
PGD (%)	4/63 (6.3)	13/208 (6.3)	4/34 (11.8)	6/113 (5.3)	1/10 (10.0)	4/81 (4.9)	0/1 (0)	2/13 (15.4)
Clinical PR (%)	37/63 (58.7)	113/208 (54.3)	19/34 (55.9)	52/113 (46.0)	5/10 (50.0)	35/81 (43.2)	1/1 (100)	7/13 (53.8)
Live birth/ongoing PR (%)	32/63 (50.8)	97/208 (46.6)	16/34 (47.1)	43/113 (38.1)	3/10 (30.0)	28/81 (34.6)	1/1 (100)	5/13 (38.5)
MGR (%)	1/37 (2.7)^b^	36/113 (31.9)^b^	0/19 (0)^c^	17/52 (32.7)^c^	0/3 (0)	12/35 (34.3)	0/1 (0)	2/7 (28.6)
Cesarean rate (%)	6/22 (27.3)	38/90(42.2)	5/13(38.5)	9/39 (23.1)	0/3 (0)	11/27 (40.7)	1/1 (100)	3/5 (60.0)
Birthweight, mean (g)	3223.4^d^	2835.0^d^	3180.9	2934.2	3345.3	2732.9	3657.1	2625.2

^a^ *P* < 0.05: one-way ANOVA.

^b^ *P* < 0.005: one-way ANOVA.

^c^ *P* < 0.01: one-way ANOVA.

^d^ *P* < 0.05: one-way ANOVA.

^e^Blastocysts: mean includes blastocysts cryopreserved and transferred.
